# Algorithm Design and Convergence Analysis for Coexistence of Cognitive Radio Networks in Unlicensed Spectrum

**DOI:** 10.3390/s23249705

**Published:** 2023-12-08

**Authors:** Yuan Zhang, Weihua Wu, Wei He, Nan Zhao

**Affiliations:** 1 State Key Laboratory of ISN, School of Telecommunications Engineering, Xidian University, Xi’an 710126, China; zhangyuan031400@hollysys.com; 2 School of Physics and Information Technology, Shaanxi Normal University, Xi’an 710119, China; whwu@snnu.edu.cn; 3 College of Electronic Countermeasures, National University of Defense Technology, Changsha 410073, China; billfovy@hotmail.com

**Keywords:** cognitive radio networks, convergence analysis, continuous-time Lyapunov drift

## Abstract

This paper focuses on achieving the low-cost coexistence of the networks in an unlicensed spectrum by making them operate on non-overlapping channels. For achieving this goal, we first give a universal convergence analysis framework for the unlicensed spectrum allocation algorithm. Then, a one-timescale iteration-adjustable unlicensed spectrum allocation algorithm is developed, where the step size and timescale parameter can be jointly adjusted based on the system performance requirement and signal overhead concern. After that, we derive the sufficient condition for the one-timescale algorithm. Furthermore, the upper bound of convergence error of the one-timescale spectrum allocation algorithm is obtained. Due to the multi-timescale evolution of the network states in the wireless network, we further propose a two-timescale iteration-adjustable joint frequency selection and frequency allocation algorithm, where the frequency selection iteration timescale is set according to the slow-changing statistical channel state information (CSI), whereas the frequency allocation iteration timescale is set according to the fast-changing local CSI. Then, we derive the convergence condition of two-timescale algorithms and the upper bound of the corresponding convergence error. The experimentalresults show that the small timescale adjustment parameter and large step size can help decrease the convergence error. Moreover, compared with traditional algorithms, the two-timescale policy can achieve throughput similar to traditional algorithms with very low iteration overhead.

## 1. Introduction

As high-speed data services continue to grow, the unlicensed spectrum has become an option for cellular operators to increase their service capabilities [1]. In total, there is over 500 MHz of spectrum bandwidth for public (e.g., (5.15, 5.25) GHz and (5.47, 5.48) GHz in the US). Compared with the licensed spectrum, the unlicensed spectrum is a completely open spectrum resource open to different operators as long as they comply with the relevant regulations. Through sharing the completely open unlicensed spectrum, operators no longer need to deploy its network resources with over-provisioning according to the peak load. In addition, it is very cost-effective to use the unlicensed spectrum. Depending on the free unlicensed spectrum resource, operators can efficiently cope with the diminishing effective revenue per GB.

Currently, WiFi is the most popular and successful wireless technology on the unlicensed bandwidths. However, the spectrum efficiency of the WiFi system is pretty low, especially when the number of subscribers is large. Hence, deploying cellular technology over the unlicensed spectrum has become a popular research topic in recent years. However, in the coexistence scenario of 5G and the WiFi network, the decision of one network can impair the decision of another markedly if they share the same unlicensed spectrum band. For example, the LTE unlicensed network (LTE-U) does not implement CSMA, whereas WiFi does. As a result, the WiFi network always senses the channel is busy until the LTE network does not have any traffic to transmit. Therefore, knowing how to achieve harmonious coexistence between LTE and incumbent systems that are already operating in unlicensed spectrum is a key challenge.

The spectrum-sharing and coexistence scenarios can be categorized into two categories, i.e., uncoordinated and coordinated schemes [2]. In the former, networks achieve coexistence based on their local information, whereas in the latter, networks should directly or indirectly exchange signals with each other. In the coordinated schemes, coordination can be implemented centralized by a cloud-based control plane via a direct communication channel [3]. With the coordination of the cloud-based control plane, heterogeneous technologies can be allocated with its corresponding clean channels. Hence, each of them can use its own resource segments without cross-tier interference. Moreover, the cloud-based control plane can also consider the load dynamics and failure scenarios of the network for adopting an adaptive coexistence scheme to avoid the underutilization of the unlicensed spectrum [4]. Due to the fact that each network can identify the existence and operation parameters of another network, coordinated schemes can provide higher performance than uncoordinated schemes. However, this performance efficiency is achieved at the expense of infrastructure/protocol complexity and coordination signal overhead. Especially when the coordination logic is deployed in a wider coverage region beyond one-hop or two-hop nodes, huge communication overhead will take place through the backhaul links.

Therefore, in this paper, we focus on achieving the low-cost coexistence of networks in the unlicensed spectrum by making them operate on separate non-overlapping channels. The use of an unlicensed spectrum allocation algorithm is critical to achieving this goal. To avoid the over-provisioning of the spectrum resource on each network, the spectrum allocation algorithm should have adaptive ability for solving the optimal solution after network state changes. However, if the spectrum allocation algorithm iteration evolves much faster than the network state dynamics, a lot of signal exchange between the coordination logic and networks will be incurred. On the contrary, if the spectrum allocation algorithm iteration evolves in a comparative timescale as the network state dynamics, the network states may have changed after a few iterations, and the existing convergence results failed to apply in this case of network states. Hence, we provide a theoretical framework in this paper for studying the convergence of the adaptive unlicensed spectrum allocation algorithm with adjustable iteration timescale. It should be noted that the theoretical framework of convergence analysis in this paper not only can be used for the coexistence of 5G and WiFi networks but also for the IoT networks with high heterogeneities.

The main contributions of this paper are summarized as follows:

- A universal convergence analysis framework is developed for the unlicensed spectrum allocation algorithm. Specifically, the algorithm iteration evolutions are modeled by a system of stochastic differential equations as a virtual dynamic system. We further reveal that the stability of the dynamic system is equivalent to the convergence of the algorithm iteration.
- A one-timescale iteration-adjustable unlicensed spectrum allocation algorithm is developed, where the step size and timescale parameter can be jointly adjusted based on the system performance requirement and signal overhead consideration. After that, we obtain the sufficient condition for the convergence of the one-timescale iteration-adjustable algorithm and the upper bound of the convergence error.
- We further propose a two-timescale iteration-adjustable joint frequency selection and frequency allocation algorithm, where the frequency selection iteration timescale is set according to the slow-changing statistical channel state information (CSI), whereas the frequency allocation iteration timescale is set according to the fast-changing local CSI. Then, we derive the convergence condition of the two-timescale algorithm and the upper bound of the corresponding convergence error under mixed timescale network states.

We organize the paper as follows. Section 2 summarizes the related work. Section 3 outlines the system model and problem formulation. Section 4 presents the static spectrum allocation iteration algorithm. Section 5 includes the one-timescale dynamic spectrum allocation algorithm. Then, in Section 6, we outline the two-timescale network state structure and develop the two-timescale dynamic spectrum allocation algorithm. In Section 7, we conduct the simulation. Section 8 concludes the paper.

## 2. Related Work

Many approaches have been proposed in these years for achieving the coexistence between WiFi and LTE-U in the unlicensed bands. These methods can be classified into the power domain approach, time domain approach and frequency domain approach.

A joint user association and power control algorithm was developed in [5] for maximizing the number of QoS-preferred users supported by LAA-LTE while protecting the WiFi users. The work in [6] proposed a power control algorithm based on Q-Learning to realize the coexistence of licensed users and unlicensed users. A stochastic optimization framework was developed in [7] for minimizing the average power consumption in the LAA-enabled SBSs and WiFi networks. To realize coexistence, the works in [8] regarded WiFi users as protected users, and then proposed a power control scheme to ensure that the interference caused by cellular users to WiFi users is less than a certain threshold. A distributed solution based on Lagrangian relaxation was proposed in [9] to assist the LTE-unlicensed network in making decisions on transmit power. Although the power domain scheme can theoretically realize the coexistence of LTE and WiFi, it is very complex to enact fine-grained control on the transmit power in real networks. On the other hand, in time-varying networks, the power control algorithm will bring huge algorithm iteration overhead and signaling transmission overhead.

The time domain scheme relies on LTE’s scheduling to periodically turn off its transmission so that WiFi users can have adequate access time [10]. Hence, the time domain scheme can be also considered as an uncoordinated scheme. The listen before talk (LBT)-based access mechanisms have been proposed in [11,12,13] for adjusting the backoff window size based on WiFi traffic load to enhance the service capacity of an LTE-U. Different from the LBT mechanism, the devices in [3,14] transmitted data on different timeslots by the polling method. However, there are many issues for using these approaches. Under the current LTE service, the time domain transmission approach degrades the rate guarantee that devices have been accustomed to. As a result, it is difficult to know why a device would select LTE service instead of using free WiFi directly. The time domain scheme usually requires all of the devices to be synchronized with each other, which would involve a major change to some LTE protocols.

Different from the time-domain scheme, in the frequency domain, the coexistence between LTE and WiFi can be achieved by having the two operate on separate, non-overlapping channels [15]. In order to achieve the frequency domain coexistence, refs. [16,17,18] developed the unlicensed spectrum splitting strategy between WiFi and the femtocell network. Note that all of these schemes need a central server to coordinate the spectrum sharing between WiFi and LTE BS, so these schemes can be considered as coordinated schemes. It should be pointed out that these schemes have no adaptive ability; the light-loaded networks cannot deliver its superfluous spectrum resource to the heavy-loaded ones. As a result, the capacity and robustness of the entire network will be seriously restrained. With this consideration, ref. [15] developed the adaptive spectrum allocation schemes for achieving the significant improvement in network performance. However, this scheme needs to reformulate a new optimization problem under the updated network states. Solving the optimization problem often needs an algorithm to be iterated many times. Hence, huge signal overhead over both the air interface and the backhaul links will be incurred. Therefore, we need to design the low-cost adaptive spectrum allocation schemes.

## 3. System Model

**Figure 1:**
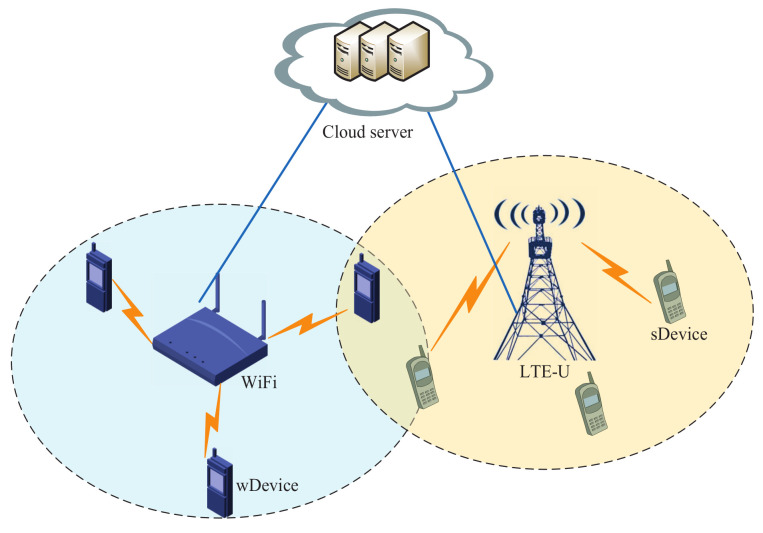
System model. The cloud server connects with LTE-U and WiFi by backhaul links. LTE and WiFi operate in different frequency bands that do not overlap each other.

LTE-U and the WiFi network are representative technologies deployed in the unlicensed spectrum. In this paper, our goal is how to realize the coexistence of LTE and WiFi. We assume that there is a cloud server deployed at the backend to implement the coordination of LTE-U and WiFi [1]. As shown in Figure 1, the cloud server connected with LTE-U and WiFi by backhaul links, and the coordinating networks have an infrastructure to exchange information about themselves or their demands. We represent the devices covered by LTE-U as sDevice, and we represent the devices covered by WiFi as wDevice. We assume that the system model in this paper consists of one WiFi, *N* LTE-U and *K* devices. Let N={1,2,…,N} denote the sets of LTE-U. Furthermore, we use K=M∪L to denote the devices set, where M and L denote the sets of wDevices and sDevices, respectively. Let *M* and *L* denote the number of devices in sets M and L. We assume that the total available bandwidth for the unlicensed spectrum is *B*.

The throughput of CSMA networks has been investigated by [19]. However, this throughput model cannot reveal the relationship between the spectrum bandwidth, channel state information and the network throughput. Different from [19], we focus more on investigating the relationship between throughput, dynamic CSI and the allocated spectrum resource. To this end, we adopt the Ideal CSMA Network (ICN) model to compute the WiFi throughput [20]. For the wDevices in set M, they compete to access the channel through CSMA protocol. We assume that each wDevice always has packets to transmit; i.e., the network operates in the saturation state. Then, the wDevice needs to spend some time on waiting, confirming and retransmitting when transmitting packets. We use $T_{tr}$ to represent the total time for transmitting a packet, which consists of packet duration, distributed Interframe Spacing, acknowledgement and short Interframe Spacing. In addition, the period of the random backoff countdown process is $T_{cd}$. Thus, the countdown overhead can be represented as $c=E\left[T_{cd}\right]/E\left[T_{tr}\right]$. The transmission state of wDevice *m* is expressed as $s_{m}$, where $s_{m}=1$ and $s_{m}=0$ indicate that the device is transmitting and waiting, respectively. After that, we use $s=s_{1}s_{2}\cdots s_{M}$ to represent the state of the network. Based on the state transition equation in [20], the stationary distribution of the state s=0⋯0 and $s=s_{1}s_{2}\cdots s_{M}$ can be given as

$$\begin{array}{c} P_{0\cdots 0}={\left(1+M/c\right)}^{-1},P_{s_{1}s_{2}\cdots s_{M}|s:s_{m}=1}={\left(c+M\right)}^{-1}. \end{array}$$

The normalized transmission rate of wDevice *m* is computed as

$$\begin{array}{c} x_{m}=P_{s_{1}s_{2}\cdots s_{M}|s:s_{m}=1}=\frac{1}{M+c}. \end{array}$$

Then, multiplying the normalized throughput by the achievable uplink transmission rate of wDevice *m*, we can obtain its actual throughput as

$$\begin{array}{c} r_{m}=\frac{\alpha_{w}}{M+c}\log_{2}\left(1+\frac{h_{m}}{\alpha_{w}}\right) \end{array}$$

where $\alpha_{w}$ denotes the fraction of the unlicensed spectrum for WiFi, $h_{m}=p_{m}{\left|g_{m}\right|}^{2}/N_{0}$, $p_{m}$ denotes the transmit power for wDevice *m*, $N_{0}$ is the power spectral density of the Gaussian background noise, and $g_{m}$ is the channel gain from wDevice *m* to WiFi. For the modest-size network, the effect of *c* on approximating the real throughput is negligible. Therefore, we give the overall throughput of the WiFi as

(1) $$\begin{array}{c} R_{WiFi}=\sum_{m=1}^{M}\frac{\alpha_{w}}{M}\log_{2}\left(1+\frac{h_{m}}{\alpha_{w}}\right) \end{array}$$

In the LTE-U network, we use $L_{n}$ to denote the set of sDevices associated with LTE-U *n*. In the proposed model, LTE-U *n* will allocate a dedicated channel $\rho_{k}^{n}$ to sDevice $k\in L_{n}$. Then, the uplink transmission rate for sDevice *k* can be computed as

(2) $$\begin{array}{c} R_{k}^{n}=\rho_{k}^{n}\log_{2}\left(1+\frac{h_{k}^{n}}{\rho_{k}^{n}}\right),\forall n\in N,k\in L_{n}. \end{array}$$

The definition of $h_{k}^{n}$ is similar to $h_{m}$.

Generally, evaluating the throughput maintained by each network is still a common approach to ensure that all collocated networks can sustain a certain level of performance [1]. Therefore, the optimization problem can be formulated as

(3) $$\begin{array}{ccc} \max & & F(\alpha_{w},\rho ;h)\\ s.t. & & \alpha_{w}+\sum_{n=1}^{N}\sum_{k\in L_{n}}\rho_{k}^{n}\leq B,\\ & & \alpha_{w}>0,\rho_{k}^{n}>0,\forall k\in L_{n},n\in N, \end{array}$$

where $F(\alpha_{w},\rho ;h)=$

$$\begin{array}{c} w_{1}\;\sum_{m=1}^{M}\;\frac{\alpha_{w}}{M}\log_{2}\left(1\;+\;\frac{h_{m}}{\alpha_{w}}\right)\;+\;w_{2}\;\sum_{n=1}^{N}\sum_{k\in L_{n}}\rho_{k}^{n}\log_{2}\left(1\;+\;\frac{h_{k}^{n}}{\rho_{k}^{n}}\right), \end{array}$$

$h={\left[h_{k}\right]}_{k\in K}$, $\rho ={\left[\rho_{k}^{n}\right]}_{k\in L}$, $w_{1}$ and $w_{2}$ are weights of throughput of sDevices and wDevices, respectively, which provide the preference of operators on wDevices or sDevices. The constraint indicates that the partitioned spectrum can’t be more than the total bandwidth.

## 4. Spectrum Allocation Iteration Algorithm

This section considers that the CSI remains unchanged before the corresponding algorithm-solving problem (3). At this case, problem (3) is a concave optimization problem. We will propose a spectrum allocation algorithm for the LTE-U/WiFi networks and then analyze the convergence of this algorithm.

### 4.1. Spectrum Allocation Iteration Algorithm

For problem (3), we construct the following Lagrange function

$$\begin{array}{c} L\left(\alpha_{w},\rho ,\lambda ;h\right)\;=\;F\left(\alpha_{w},\rho ;h\right)\;-\;\lambda \;\left(\;\alpha_{w}\;+\;\sum_{n=1}^{N}\sum_{k\in L_{n}}\rho_{k}^{n}\;-\;B\;\right)\;, \end{array}$$

where λ is the Lagrange multiplier. Then, the following Karush-Kuhn-Tucker (KKT) conditions for problem (3) are given as

(4) $$\begin{array}{c} \frac{\partial L(\alpha_{w},\rho ,\lambda ;h)}{\partial \alpha_{w}}=0, \end{array}$$

(5) $$\begin{array}{c} \frac{\partial L(\alpha_{w},\rho ,\lambda ;h)}{\partial \rho_{k}^{n}}=0,\forall n\in N,k\in L_{n}, \end{array}$$

(6) $$\begin{array}{c} \lambda \;\left(\;\alpha_{w}\;+\;\sum_{n=1}^{N}\sum_{k\in L_{n}}\rho_{k}^{n}\;-\;B\;\right)=0. \end{array}$$

For convenience, we express the spectrum allocation variables $\alpha_{w}$,ρ and λ as $x=\left(\alpha_{w},\rho ,\lambda \right)\in R_{+}^{L+2}$. Therefore, at the $n_{s}$-th solt, x is iterated as

(7) $$\begin{array}{c} x_{n_{s}+1}=\Gamma_{+}\left[x_{n_{s}}+\gamma_{n_{s}}G\left(x_{n_{s}};h_{n_{s}}\right)\right], \end{array}$$

where $\gamma_{n_{s}}$ is the step size, and $\Gamma_{+}$ denotes the projection onto the non-negative domain. The mapping of G(·) is given as

(8) $$\begin{array}{c} G\left(\cdot \right)=\left(\begin{array}{c} \frac{\partial F(\alpha_{w},\rho ;h)}{\partial \alpha_{w}}-\lambda \\ \frac{\partial F(\alpha_{w},\rho ;h)}{\partial \rho }-\lambda \\ \;\alpha_{w}\;+\;\sum_{n=1}^{N}\sum_{i\in L_{n}}\rho_{i}^{n}\;-\;B\; \end{array}\right)\in R_{+}^{L+2}. \end{array}$$

Although many works have proved the convergence of the iteration algorithm [21,22], it is difficult to apply them directly in the scenario where the CSI is time-varying. In a dynamic wireless network, when the network state changes, the optimal spectrum allocation solution will also change, which may result in existing convergence results failling to apply. Hence, in the following, we will explore the convergence performance of the spectrum allocation algorithm in static LTE/WiFi networks. This study will provide a basis for analyzing the convergence of spectrum partitioning algorithms in dynamic network environments.

### 4.2. Convergence Analysis

If we regard the spectrum allocation solution x as a dynamic system evolving with the change in network state h, then the state trajectory x can be considered as the solution to the following equation:

(9) $$\begin{array}{c} dx=\gamma G(x;h)dt, \end{array}$$

where the subscript of γ is omitted for the sake of convenience.

Then, the equilibrium point of a specific dynamic system can be defined as

**Definition** **1.** (Equilibrium Point): The solution $x^{*}$ satisfying $G(x^{*};h)=0$ is defined as the equilibrium point.

In static wireless networks, in the iterative process of the algorithm, CSI h is always unchanged. Hence, it should be noted that the equilibrium point $x^{*}$ is fixed. In order to analyze the convergence performance of the algorithm, we give the definition of convergence error.

**Definition** **2.** (Convergence Error): Convergence error of the spectrum allocation algorithm is defined as the error between the iterative output x and the optimality $x^{*}$, i.e., $x_{e}=x-x^{*}$.

Then, based on this definition, the differential equation of convergence error is defined as

(10) $$\begin{array}{c} dx_{e}=dx-dx^{*}=\gamma G\left(x;h\right)dt. \end{array}$$

Equation (10) can be also considered as a dynamic system. Therefore, the convergence of the algorithm in Equation (7) is equivalent to the asymptotic stability of the dynamic system defined in Equation (10).

Then, we use Lyapunov theory to study the stability of the system. First, we define a Lyapunov function as $V=\frac{1}{2}x_{e}^{T}x_{e}$. Motivated from the definition of Lyapunov drift in a discrete-time system, the Lyapunov drift in a continuous-time dynamic system is defined as

(11) $$\begin{array}{c} LV\left(x_{e}\right)=\lim_{\delta \to 0}\frac{E[V\left(x_{e}\left(t+\delta \right)\right)-V\left(x_{e}\left(t\right)\right)|x_{e}\left(t\right)]}{\delta }. \end{array}$$

Lyapunov drift indicates the growth direction of the Lyapunov function $V(x_{e})$ over time, which can be also considered as an energy function. Obviously, the negative Lyapunov drift contributes to the stability of the system. However, Equation (11) is not easy to calculate. In order to obtain an accurate expression, the Lyapunov drift $LV(x_{e})$ can also be calculated by the following lemma.

**Lemma** **1.** *(Continuous-Time Lyapunov Drift [23]): Suppose that there is a stochastic process z described as*

(12) $$\begin{array}{c} dz=f(z)dt+g(z)dW, \end{array}$$

*the stochastic Lyapunov drift of V(z) can be written as*

$$\begin{array}{c} LV\left(z\right)=\frac{\partial V(z)}{\partial z}f\left(z\right)+tr\left[g{\left(z\right)}^{T}\frac{\partial^{2}V\left(z\right)}{\partial z\partial z^{T}}g\left(z\right)\right], \end{array}$$

*where W is a standard complex Wiener process.*

Lemma 1 builds a bridge between the dynamic system in Equation (10) and the Lyapunov drift. Therefore, in the following, we will study the stability of dynamic systems by exploring the characteristics of Lyapunov drift.

**Lemma** **2.** *Suppose that the Lyapunov drift of V(z) satisfies*

$$\begin{array}{c} V(z)\leq -a∥z∥+g(s), \end{array}$$

*then z(t) is stable and satisfies*

$$\begin{array}{c} \lim\underset{t\to \infty }{\sup}\frac{1}{t}\int_{0}^{t}E\left[z\left(\tau \right)\right]d\tau \leq \frac{d}{a}. \end{array}$$

*where s(t) satisfies $\lim\sup_{t\to \infty }\frac{1}{t}\int_{0}^{t}E\left[g\left(s\left(\tau \right)\right)\right]d\tau \leq d$ for d<∞ and a is a positive constant.*

**Proof.**  The similar proof is shown in [24]. □

In Lemma 2, $\frac{d}{a}$ can be considered as an estimation on the upper bound of convergence error. The advantage of Lemma 2 is that it provides a qualitative analysis of the dynamic system. Then, based on Lemma 2, the Lyapunov drift of the dynamic system in Equation (10) can be computed as shown in Equation (13).

(13) $$\begin{array}{ccc} LV(x_{e})\;= & & \;\;\;\;x_{e}^{T}\gamma G\left(x;h\right)\;=\;\gamma \left(\alpha_{w}-\alpha_{w}^{*}\right)\left(\frac{\partial F(\cdot )}{\partial \alpha_{w}}\;-\;\lambda \right)\;+\;\gamma \sum_{n=1}^{N}\sum_{k\in L_{n}}\left(\rho_{k}^{n}\;-\;\rho_{k}^{n,*}\right)\left(\frac{\partial F(\cdot )}{\partial \rho_{k}^{n}}\;-\;\lambda \right)\;\\ & & \;+\;\gamma \left(\lambda \;-\;\lambda^{*}\right)\left(\;\alpha_{w}\;+\;\sum_{n=1}^{N}\sum_{k\in L_{n}}\rho_{k}^{n}\;-\;B\;\right)\\ & & =\gamma \left(\alpha_{w}-\alpha_{w}^{*}\right)\left(\frac{\partial F(\cdot )}{\partial \alpha_{w}}-\frac{\partial F(\cdot )}{\partial \alpha_{w}^{*}}\right)+\gamma \left(\alpha_{w}-\alpha_{w}^{*}\right)\left(\frac{\partial F(\cdot )}{\partial \alpha_{w}}-\lambda^{*}\right)\\ & & \;+\gamma \sum_{n=1}^{N}\sum_{k\in L_{n}}\left(\rho_{k}^{n}-\rho_{k}^{n,*}\right)\left(\frac{\partial F(\cdot )}{\partial \rho_{k}^{n}}-\frac{\partial F(\cdot )}{\partial \rho_{k}^{n,*}}\right)\\ & & \;+\gamma \sum_{n=1}^{N}\sum_{k\in L_{n}}\left(\rho_{k}^{n}-\rho_{k}^{n,*}\right)\left(\frac{\partial F(\cdot )}{\partial }\rho_{k}^{n}-\lambda^{*}\right)+\left(\lambda -\lambda^{*}\right)\left(\alpha_{w}^{*}+\sum_{n=1}^{N}\sum_{k\in L_{n}}\rho_{k}^{n,*}-B\right)\\ & & \leq \gamma \left(\alpha_{w}-\alpha_{w}^{*}\right)\left(\frac{\partial F(\cdot )}{\partial \alpha_{w}}-\frac{\partial F(\cdot )}{\partial \alpha_{w}^{*}}\right)+\gamma \sum_{n=1}^{N}\sum_{k\in L_{n}}\left(\rho_{k}^{n}-\rho_{k}^{n,*}\right)\left(\frac{\partial F(\cdot )}{\partial \rho_{k}^{n}}-\frac{\partial F(\cdot )}{\partial \rho_{k}^{n,*}}\right)\leq -\alpha_{x}\gamma {∥x_{e}∥}^{2}. \end{array}$$

In Equation (13), the first inequality is based on the KKT conditions in Equations (5) and (6), the second inequality is because F(·) is a concave function.

It can be easily seen that the Lyapunov drift in Equation (13) satisfies the stochastically stable condition in Lemma 2. Hence, we can conclude that the algorithm can iterate to the optimal value and that the convergence error is zero, i.e., $\lim\sup_{t\to \infty }\frac{1}{t}\int_{0}^{t}E\left[x_{e}\left(\tau \right)\right]d\tau =0$.

However, due to the constant step size, the iteration algorithm definitely converges to within some range of the optimal value; i.e., we have $\lim_{n_{s}\to \infty }\left|F_{bset}^{n_{s}}-F^{*}\right|\leq \frac{\gamma \epsilon^{2}}{2}$, where $F^{*}$ denotes the optimal value problem (3), $F_{bset}^{n_{s}}$ is the best objective value found in $n_{s}$ iterations, and ϵ is the upper bound of the norm of G [22]. We refer to this part of error as the steady-state error. As a result, the convergence of the spectrum allocation iteration algorithm can be measured by the convergence error and steady-state error. The former determines whether the algorithm is convergent, and the latter shows the quality of the convergence result.

### 4.3. Signaling Overhead

The spectrum allocation algorithm should be executed with the coordination of the centralized cloud server. Although the coordinated scheme provides higher performance, the coordination overhead will be increased sharply. Especially for the heterogeneous LTE-U and WiFi network, a cross-technology communication channel is used for exchanging the corresponding information [25]. In order to implement the spectrum allocation, the cloud server needs to acquire all of the CSI at each algorithm iteration. Let $c_{1}$ and $c_{2}$ denote the signaling overheads on wireless links and backhaul links, respectively. In order to achieve δ-optimality, i.e., $|\lambda -\lambda^{*}|\leq \delta$, it needs the order of $O(\frac{1}{\delta^{2}})$ iterations to solve the system through a maximization problem. Hence, the amount of signaling overhead between the cloud server and the devices is $O(\frac{K(c_{1}+c_{2})}{\delta^{2}})$.

**Remark** **1.** *It is certainly true that the iteration algorithm can converge to the optimal value. However, we can observe from Equation (11) that the spectrum partitioning decision of the WiFi network depends on the statistical global CSI. It is not difficult to understand that the statistical global CSI is affected by the large-scale channel fading, whose statistical properties change over a relatively large timescale. In contrast, the spectrum partitioning decision of LTE user is affected by the local CSI. Moreover, the local CSI changes on a small timescale. Therefore, if the spectrum partitioning decisions of the LTE and WiFi are iterated at the same time on a smaller timescale, although the optimal solution can be found, the iteration of WiFi will bring additional iteration overhead and signaling overhead. In contrast, if we iterate the two decisions on a large timescale, the spectrum partitioning decision of the LTE will waste many transmission opportunities on the small timescale. Based on this motivation, we will first explore the convergence of an one-timescale algorithm and extend its theoretical framework to the two-timescale algorithm.*

## 5. One-Timescale Dynamic Spectrum Allocation

**Figure 2:**
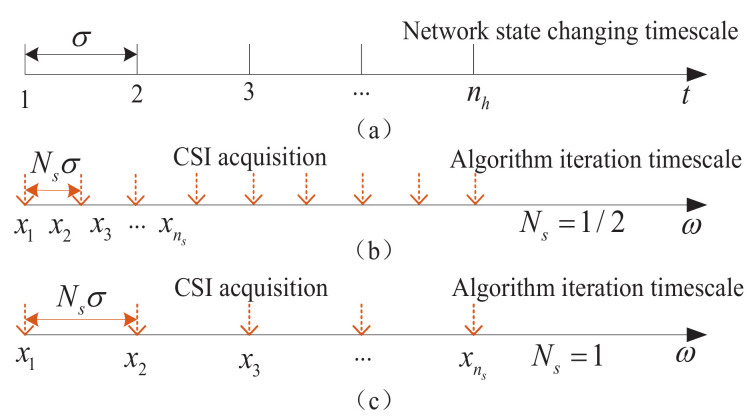
Network state changing timescale vs. one-timescale algorithm iteration.

Obviously, the static CSI assumption during the algorithm iteration in the above section is not practical. In fact, the CSI is dynamic and behaves as a stochastic process, along with the spectrum allocation algorithm iteration. In this situation, a fast iterative algorithm, as shown in Figure 2b, may converge to the moving optimal solution. However, the complexity and coordination overhead may be too large to undertake. As shown in Figure 2c, the iteration number and signal overhead will reduce with the increase in the algorithm iteration timescale. However, the convergence of the algorithm cannot be ensured. With this consideration, in this section, we will explore whether the algorithm can converge in dynamic environments and its convergence error.

### 5.1. One-Timescale Algorithm

In dynamic wireless networks, the fading of wireless links satisfies Rayleigh distribution. The signal incoming to the receiver contains a large number of reflected radio waves, which are characterized by “multipath reception”. Thus, the wireless channel behaves in a random-like fashion, and its random behavior can be described by the following stochastic equation [26],

(14) $$\begin{array}{c} dh=-\frac{1}{2}a_{h}hdt+a_{h}^{\frac{1}{2}}dW_{K}, \end{array}$$

where $W_{K}$ denotes the *K*-dimensional Wiener process and $a_{h}$ is the temporal correlation of h.

Without loss of generality, we assume that the CSI is changing at the slotted time and the slot length is represented as sufficient constant σ. Then, the slot length of the iteration algorithm can be set as $N_{s}\sigma$, where $N_{s}$ is the timescale adjustment parameter, and it can be continuously taken values in (0,1]. As shown in Figure 2, a small $N_{s}$ indicates more iterations of the spectrum allocation algorithm along with the CSI changing. Therefore, at the $n_{s}$-th slot, the spectrum allocation variable x can be also updated according to the following iterations

(15) $$\begin{array}{c} x_{n_{s}+1}=\Gamma_{+}\left[x_{n_{s}}+\gamma_{n_{s}}G\left(x_{n_{s}};h_{n_{s}}\right)\right]. \end{array}$$

Equation (15) characterizes a discrete iterative algorithm. To describe its iterative trajectory, we construct the following dynamic system

(16) $$\begin{array}{c} dx=\gamma G(x;h)d\omega , \end{array}$$

where ω is the timescale of the spectrum allocation algorithm.

In order to analyze the convergence of the algorithm in the network, we need to relate the algorithm iteration to the running time of the network. From the viewpoint of algorithm iteration, the length of each iteration is σ; thus, the length of $n_{s}$ iterations can be expressed as the real number ω, i.e., $\omega =n_{s}\sigma$. From the viewpoint of the network running, the time of an single algorithm iteration is $\sigma N_{s}$. Thus, after $n_{s}$ iterations, the running time of the network is $t=n_{s}\sigma N_{s}$. After the above analysis, we found that $n_{s}=\omega /\sigma =t/\left(\sigma N_{s}\right)$. Hence, we can obtain the relation between ω and *t* as

(17) $$\begin{array}{c} t=\omega N_{s},dt=N_{s}d\omega . \end{array}$$

Based on the equation above, the dynamic system can be mapped from ω to *t* as

(18) $$\begin{array}{c} dx=\gamma G\left(x;h\right)d\omega =\gamma N_{s}^{-1}G\left(x;h\right)dt. \end{array}$$

### 5.2. Convergence Analysis

In a dynamic wireless network, the optimal solution, i.e., equilibrium point, is always disturbed by the external stochastic CSI processes. Therefore, the equilibrium point is also a stochastic process. Based on the optimality condition G(·)=0 and the implicit theorem, the dynamic of the equilibrium point can be computed as

(19) $$\begin{array}{c} dx^{*}=-G_{x^{*}}^{-1}G_{h}dh, \end{array}$$

where $G_{x^{*}}≜\frac{\partial }{\partial x^{*}}G\left(\cdot \right)$, $G_{h}≜\frac{\partial }{\partial h}G\left(\cdot \right)$.

Then, by subtracting Equation (19) from Equation (18), we can obtain the convergence error for the algorithm iteration.

(20) $$\begin{array}{c} dx_{e}=dx-dx^{*}=\gamma N_{s}^{-1}G\left(\cdot \right)dt-G_{x^{*}}^{-1}G_{h}dh. \end{array}$$

By substituting Equation (14) into Equation (20), the convergence error can be further computed as follows

(21) $$\begin{array}{c} dx_{e}\;=\;\left(\;\gamma N_{s}^{-1}G\left(\cdot \right)\;+\;\frac{1}{2}aG_{x^{*}}^{-1}G_{h}h\;\right)dt\;-\;a^{\frac{1}{2}}G_{x^{*}}^{-1}G_{h}dW_{K}. \end{array}$$

We use $z=\left(x_{e},h\right)\in R^{L+2+K}$ to represent the state of a joint system. Then, Equations (14) and (21) can be used to construct the following dynamic system

(22) $$\begin{array}{c} dz=U_{1}\left(z\right)dt+U_{2}\left(z\right)d\tilde{W}, \end{array}$$

where

$$\begin{array}{c} U_{1}\;=\;\left[\;\;\begin{array}{c} \gamma N_{s}^{-1}G\left(\cdot \right)\;+\;\frac{1}{2}aG_{x^{*}}^{-1}G_{h}h\\ -\frac{1}{2}ah \end{array}\;\;\right],U_{2}\;=\;\left[\;\;\begin{array}{c} -a^{\frac{1}{2}}G_{x^{*}}^{-1}G_{h}\\ a^{\frac{1}{2}}I_{K} \end{array}\;\;\right], \end{array}$$

$\tilde{W}$ is a (L+2+K)-dimensional complex Wiener process.

Here, we find that the dynamic system is driven by the complex Wiener process $\tilde{W}$. Hence, we can analyze the convergence of the spectrum allocation algorithm by studying the stability of the dynamic system. Motivated from this consideration, we first define a Lyapunov function of z as $V=\frac{1}{2}z^{T}z$. Similarly to the convergence analysis in Section 3, the Lyapunov drift of the dynamic system in (22) can be computed according to Lemma 1 as

(23) $$\begin{array}{ccc} LV(z)\;\;\;\;\;\;\;\;\; & & =z^{T}U_{1}\left(z\right)+\mathrm{tr}\left[U_{2}{\left(z\right)}^{T}U_{2}\left(z\right)\right]\\ & & \leq x_{e}^{T}\left(\gamma GN_{s}^{-1}+\frac{1}{2}aG_{x^{*}}^{-1}G_{h}h\right)\\ & & \;+a\mathrm{tr}\left[{\left(G_{x^{*}}^{-1}G_{h}\right)}^{T}\left(G_{x^{*}}^{-1}G_{h}\right)\right]\;-\;\frac{1}{2}h^{T}h\;+\;aK. \end{array}$$

In Equation (23), except for the network state parameters, such as *a* and h, the step size γ and the timescale parameter $N_{s}$ dominate the value of the Lyapunov drift. Based on Lemma 2, the dynamic system will be stable if the Lyapunov drift can be upper bounded by a negative function. Hence, by making the Lyapunov drift satisfying the stability constraint in Lemma 2, we can obtain the following theorem.

**Theorem** **1.** (Convergence of the one-timescale algorithm): Suppose that there exists $0<v_{xh}<\infty$, such that $∥G_{x^{*}}^{-1}G_{h}∥\leq v_{xh}$. Then, if the step size parameter γ and timescale parameter $N_{s}$ satisfy

$$\begin{array}{c} \frac{\gamma }{N_{s}}\geq \frac{av_{xh}^{2}}{8\alpha_{x}}, \end{array}$$

*the one-timescale algorithm can converge to the moving optimal solution.*

**Proof.**  First of all, from the convergence analysis in Equation (13), we can obtain $x_{e}^{T}G\leq -\alpha_{x}{∥x_{e}∥}^{2}$. Then, using this result and the assumption in **Theorem 1**, the upper bound of the Lyapunov drift can be computed as

$$\begin{array}{ccc} LV(z)\;\;\;\;\;\;\;\; & & \leq -\alpha_{x}\gamma N_{s}^{-1}∥x_{e}{∥}^{2}-\frac{1}{2}{a∥h∥}^{2}+\frac{1}{2}av_{xh}∥h∥∥x_{e}∥\\ & & +av_{xh}^{2}K+aK=-\chi^{T}A\chi +av_{xh}^{2}N_{h}+aN_{h}, \end{array}$$

where $\chi =[∥x_{e}∥,∥h∥]$ and

$$\begin{array}{c} A=\left[\begin{array}{cc} \alpha_{x}\gamma N_{s}^{-1} & -\frac{1}{4}av_{xh}\\ -\frac{1}{4}av_{xh} & \frac{1}{2}a \end{array}\right]. \end{array}$$

Obviously, when the coefficient matrix A is positive, the dynamic system is stable. Therefore, by making the matrix positive definite, the condition in Theorem 1 can be obtained. □

In addition to the stability, the convergence error is another metric to show the convergence performance of the one-timescale algorithm.

**Theorem** **2.** *(Upper Bound of Convergence Error): The convergence error of the one-timescale algorithm can be given as*

$$\begin{array}{c} \eta \leq {\left(\frac{N_{s}}{\gamma }\right)}^{2}{\left(\frac{av_{xh}\pi }{2\alpha_{x}}\right)}^{2}+\frac{N_{s}}{\gamma }\frac{2av_{xh}^{2}K}{\alpha_{x}} \end{array}$$

**Proof.**  Following from Equation (21), the Lyapunov drift of $x_{e}$ can be calculated based on Lemma 1 as

$$\begin{array}{ccc} LV(x_{e})\;\;\;\;\;\;\;\; & & \leq x_{e}^{T}\left(\gamma GN_{s}^{-1}+\frac{1}{2}aG_{x^{*}}^{-1}G_{h}h\right)\\ & & \;+a\mathrm{tr}\left[{\left(G_{x^{*}}^{-1}G_{h}\right)}^{T}\left(\;G_{x^{*}}^{-1}G_{h}\right)\right]\\ & & \leq -\alpha_{x}\gamma N_{s}^{-1}∥x_{e}{∥}^{2}+\frac{1}{2}av_{xh}\pi ∥x_{e}∥+av_{xh}K\\ & & \leq -\frac{1}{2}\alpha_{x}\gamma N_{s}^{-1}{∥x_{e}∥}^{2}+av_{xh}^{2}K+\frac{{\left(av_{xh}\pi \right)}^{2}}{8\alpha_{x}\gamma N_{s}^{-1}} \end{array}$$

where $\pi =\max_{k\in K}\left\{h_{k}\right\}$ is the maximum channel gains among *K* transmission links. According to Lemma 2, the convergence error can be obtained as shown in Theorem 2. □

The above results motivate the following remark on the convergence and convergence error.

In Theorems 1 and 2, *a* specifies the changing speed of the wireless CSI, *K* can be interpreted as the network size, $v_{xh}$ represents the sensitivity of the equilibrium point corresponding to the time-varying CSI and $\alpha_{x}$ is the convergence rate of the spectrum allocation problem. Therefore, for the given network situation, i.e., (a,K), we can increase the iteration number ($N_{s}$) or set a large step size (γ) for achieving the algorithm convergence and decreasing the convergence error for the certain spectrum allocation algorithm ($\alpha_{x}$,$v_{xh}$). On the other hand, for the given spectrum allocation algorithm ($\alpha_{x}$,$v_{xh}$) and adjustment parameters $\left(\gamma ,N_{s}\right)$, the CSI changing speed *a* not only affects the convergence but also determines the convergence error. However, the network size *K* only determines the convergence error, which comes from the accumulation of convergence error on each link.

### 5.3. Signaling Overhead

For the one-timescale spectrum allocation algorithm, it needs the order of $O(1/N_{s})$ iterations at each slot of the CSI. Similar to the signal overhead in Section 4, at each iteration, the cloud server needs to acquire all of the CSI. Therefore, the signaling overhead amount between the cloud server and devices is $O(\frac{K(c_{1}+c_{2})}{N_{s}})$, which is much smaller than the signaling overhead in Section 4.

## 6. Two-Timescale Dynamic Spectrum Allocation

In above section, we observe that the fraction of the unlicensed spectrum for WiFi $\alpha_{w}$ adapts to the CSI statistics $h_{m},m\in M$ with a global coordination, while the unlicensed spectrum allocation $\rho_{k}^{n}$ should adapt to the instantaneous CSI $h_{k}^{n}$ locally. In fact, In fact, obtaining real-time local CSI is practical, while obtaining real-time global CSI is extremely difficult [27]. Therefore, the one-timescale spectrum allocation algorithm not only is difficult to achieve in practice but also has very sensitive system performance due to signaling latency in acquiring the global CSI. In this section, we will decompose the spectrum allocation into a short-term control and a long-term control. Furthermore, by iterating them on different timescales, the spectrum allocation policy can not only exploit the instantaneous transmission opportunity at small timescale but also save a lot of signal overhead at the large timescale.

### 6.1. Two-Timescale Algorithm Dynamics

Motivated from the above discussion, we employ the mixed timescale CSI model, i.e., $h_{k}=h_{k}^{l}h_{k}^{s}$, where $h_{k}^{l}$ is the large timescale fading and $h_{k}^{s}$ is the small timescale fading [27,28]. Furthermore, the dynamics of $h_{k}^{l}$ and $h_{k}^{s}$ can be specified by the following stochastic differential equations:

(24) $$\begin{array}{ccc} & & dh_{k}^{s}=-\frac{1}{2}ah_{k}^{s}dt+a^{\frac{1}{2}}dW,\forall k\in K, \end{array}$$

(25) $$\begin{array}{ccc} & & dh_{k}^{l}=-c_{0}D_{k}{\left(t\right)}^{-\iota -1}v_{k}\left(t\right)dt,\forall k\in K, \end{array}$$

where $c_{0}$ is an antenna-gain-related constant, $D_{j}\left(t\right)\geq D_{min}$ is the distance between device *k* and the corresponding network, $v_{k}\left(t\right)$ is the relative moving speed of device *k*, and ι is the path loss exponent.

In fact, in any form of wireless network, obtaining real-time global CSI from a central node is extremely difficult and expensive. Hence, we assume that only the network, WiFi or LTE-U, has the knowledge of the local CSI $\left(h_{k}^{l},h_{k}^{s}\right)$ of the connected devices at the small timescale. On the other hand, the cloud entity can only acquire the global CSI $h_{k}^{l}$ for all of the devices at the large timescale. Therefore, the spectrum allocation can be achieved at two-timescales. At the large timescale, the cloud entity select the clean block of bandwidth for WiFi or LTE-U based the global CSI. On the small timescale, the frequency allocation can be executed in the selected frequency by LTE-U using the local CSI.

Let $B_{n}$ denote the selected clean block of bandwidth for LTE-U *n*, and let $\rho_{k}^{n}$ denote the allocated frequency for sDevice *k*. We can obtain the following constraint

$$\begin{array}{c} \alpha_{w}+\sum_{n=1}^{N}B_{n}\leq B_{max},\sum_{k\in L_{n}}\rho_{k}^{n}\leq B_{n}, \end{array}$$

where the first equation indicates that the selected bandwidth for WiFi and LTE-U should be smaller than the total unlicensed spectrum and the second equation indicates the frequency allocation constraint for LTE-U.

Then, the two-timescale spectrum allocation problem can be formulated as

(26) $$\begin{array}{ccc} \max & & E[F\left(\alpha_{w},\rho ,B,h\right)]\\ s.t. & & \alpha_{w}+\sum_{n=1}^{N}B_{n}\leq B,\\ & & \sum_{l\in L_{n}}\rho_{n}^{l}\leq B_{n},\\ & & \alpha_{w}>0,B_{n}>0,\rho_{k}^{n}>0,\forall n\in N,k\in K. \end{array}$$

In problem (26), the constraints are coupled by the frequency selection variable $B_{n}$. Hence, when the variable $B_{n}$ is fixed, the problem would decouple. Therefore, it makes sense to decompose problem (26) into two levels of optimization. At the lower level, we have the frequency allocation subproblems for LTE-U *n* as follows

(27) $$\begin{array}{ccc} F_{n}\left(B_{n},h_{n}\right)=\max\;\;\;\;\;\;\; & & \;w_{2}\sum_{k\in L_{n}}\;\rho_{k}^{n}\log_{2}\left(1+\frac{h_{k}^{n}}{\rho_{k}^{n}}\right)\\ s.t.\;\;\;\;\;\;\; & & \sum_{k\in L_{n}}\rho_{k}^{n}\leq B_{n},\rho_{k}^{n}>0,\forall k\in L_{n}, \end{array}$$

where $h_{n}={\left[h_{k}^{n}\right]}_{k\in L_{n}}$. At the higher level, we have the master problem in charge of solving the frequency selection problem.

(28) $$\begin{array}{ccc} \max\;\;\;\;\; & & E\;\left[\;w_{1}\;\;\;\sum_{m=1}^{M}\;\frac{a}{M}\alpha_{w}\log_{2}\left(\;1\;+\;\frac{g_{m}}{\alpha_{w}}\;\right)\;\;+\;\;\sum_{n=1}^{N}\;F_{n}\;\left(B_{n},h_{n}\right)\;\right]\\ s.t.\;\;\;\; & & \alpha_{w}+\sum_{n=1}^{N}B_{n}\leq B,\alpha_{w}>0,B_{n}>0,\forall n\in N. \end{array}$$

**Figure 3:**
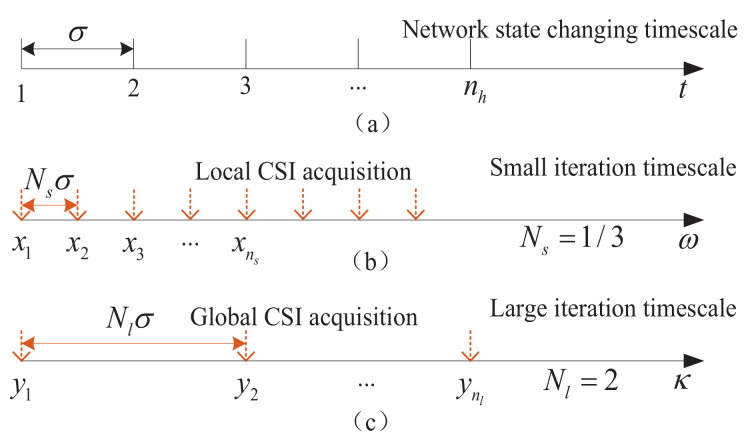
Network state changing timescale vs. two-timescale algorithm iteration.

Let $x=\left(\rho ,\lambda \right)\in R_{+}^{L+1}$ be the small timescale frequency allocation variable of the lower level subproblem. Similarly, let $y=\left(\alpha_{w},B,\mu \right)\in R_{+}^{N+2}$ be the large timescale frequency selection variable of the master problem. In x and y, both λ and μ are the lagrange multiplier variables. As shown in Figure 3, the slot length of CSI is σ. The slot length of the small timescale iteration algorithm is set as $N_{s}\sigma$, where $N_{s}$ is the small timescale adjustment parameter, and it can be continuously taken values in (0,1]. On the other hand, the slot length of the large timescale iteration is $N_{l}\sigma$, where $N_{l}\geq 1$ is the large timescale adjustment parameter. Let $n_{s}$ and $n_{l}$ denote the slot index of the small timescale iteration and large timescale iteration, respectively. The frequency selection and frequency allocation can be updated according to the following iterations

(29) $$\begin{array}{ccc} y_{n_{l}}\;\;\;\;\;\; & & =\Gamma_{+}\left[y_{n_{l}-1}+\mu K\left(y_{n_{l}-1};h_{n_{l}-1}^{l}\right)\right], \end{array}$$

(30) $$\begin{array}{ccc} x_{n_{s}}\;\;\;\;\;\; & & =\Gamma_{+}\left[x_{n_{s}-1}+\gamma G\left(x_{n_{s}-1},y_{n_{l}};h_{n_{s}-1}^{s},h_{n_{l}}^{l}\right)\right]. \end{array}$$

Then, the trajectories of the algorithm iteration in Equations (29) and (30) can be characterized by the following dynamic system

(31) $$\begin{array}{c} dy=\mu K\left(y;h^{l}\right)d\kappa ,dx=\gamma G\left(x,y;h^{s},h^{l}\right)d\omega , \end{array}$$

where κ and ω are the virtual timescales of frequency selection and frequency allocation, respectively. Under the continuous-time algorithm, the iteration indices of the two-timescale algorithm are represented as $n_{s}=\left\lfloor \frac{\omega }{N_{s}\sigma }\right\rfloor$, $n_{l}=\left\lfloor \frac{\kappa }{N_{l}\sigma }\right\rfloor$. Then, based on the relation with the CSI timescale, i.e., $n_{h}=\left\lfloor \frac{t}{\sigma }\right\rfloor$, the timescale relation of κ, ω and *t* is given as

$$\begin{array}{c} t=\kappa N_{l}^{-1},t=\omega N_{s}^{-1},dt=N_{l}^{-1}d\kappa ,dt=N_{s}^{-1}d\omega . \end{array}$$

Then, the dynamics of the wireless network under frequency selection and frequency allocation algorithms are determined by the following coupled equations

(32) $$\begin{array}{ccc} & & dh=-\frac{1}{2}ahdt+a^{\frac{1}{2}}dW, \end{array}$$

(33) $$\begin{array}{ccc} & & dx=\gamma N_{s}^{-1}G\left(x,y;h^{s},h^{l}\right)dt, \end{array}$$

(34) $$\begin{array}{ccc} & & dy=\gamma N_{l}^{-1}K\left(y;h^{l}\right)dt. \end{array}$$

The equilibrium point is a stochastic point driven by external conditions. Therefore, it is necessary to study whether the iteration of the algorithm can track the change in the above equilibrium point and its tracking error.

### 6.2. Convergence Analysis

For the two-timescale iteration algorithms, the trajectory of equilibrium $y^{*}\left(h^{l}\right)$ is driven by the time-varying $h^{l}$. Due to the slow variation of $h^{l}$, the trajectory of equilibrium $x^{*}\left(y,h^{s},h^{l}\right)$ is mainly driven by the time-varying y and $h^{s}$. According to the implicit theorem and the optimality conditions G(·)=0,K(·)=0, we can obtain the following equations

(35a) $$\begin{array}{ccc} & & dx^{*}\;\;=\;-G_{x^{*}}^{-1}G_{h^{s}}dh^{s}\;-\;G_{x^{*}}^{-1}G_{y}dy, \end{array}$$

(35b) $$\begin{array}{ccc} & & dy^{*}\;\;=\;-K_{y^{*}}^{-1}K_{h^{l}}dh^{l}, \end{array}$$

where $G_{x^{*}}≜\frac{\partial }{\partial x^{*}}G\left(\cdot \right)$, $G_{h^{s}}≜\frac{\partial }{\partial h^{s}}G\left(\cdot \right)$, $G_{y}≜\frac{\partial }{\partial y}G\left(\cdot \right)$ and $K_{y^{*}}≜\frac{\partial }{\partial y^{*}}K\left(\cdot \right)$, $K_{h^{l}}≜\frac{\partial }{\partial h^{l}}K\left(\cdot \right)$. Then, the convergence error can be computed as

(36) $$\begin{array}{ccc} \;dx\;-\;dx^{*}\;\;\;\;\;\;\; & & =\gamma N_{s}^{-1}G\left(\cdot \right)dt+G_{x^{*}}^{-1}G_{h^{s}}dh^{s}\;\;+\;\;G_{x^{*}}^{-1}G_{y}dy, \end{array}$$

(37) $$\begin{array}{ccc} dy\;-\;dy^{*}\;\;\;\;\; & & =\;\gamma N_{l}^{-1}K\left(\cdot \right)dt\;+\;K_{y^{*}}^{-1}K_{h^{l}}dh^{l}. \end{array}$$

Substituting Equations (24) and (25) into the above equations, we obtain

(38) $$\begin{array}{ccc} dx_{e}\;\;\;\;\;\;\;\;\;\;\; & & =\left(\gamma N_{s}^{-1}G\left(\cdot \right)+\gamma N_{l}^{-1}G_{x^{*}}^{-1}G_{y}K\left(\cdot \right)\right.\\ & & \;\left.-\frac{1}{2}aG_{x^{*}}^{-1}G_{h^{s}}h^{s}\right)dt+a^{\frac{1}{2}}G_{x^{*}}^{-1}G_{h^{s}}dW, \end{array}$$

(39) $$\begin{array}{ccc} dy_{e}\;\;\;\;\;\; & & =\left(\gamma N_{l}^{-1}K\left(\cdot \right)+K_{y^{*}}^{-1}K_{h^{l}}H_{L}\right)dt \end{array}$$

where $H_{L}$ is a diagonal matrix, and $H_{L}\left(i,i\right)=c_{0}D_{i}{\left(t\right)}^{-\iota -1}v_{i}\left(t\right)$. Let $u=(x_{e},y_{e},h^{s})$ be a joint system state. Then, Equations (38) and (39) can construct the following dynamic system

(40) $$\begin{array}{c} du=U_{1}\left(u\right)dt+U_{2}\left(u\right)d\ddot{W}, \end{array}$$

where

$$\begin{array}{c} U_{1}=\left[\begin{array}{c} \gamma N_{s}^{-1}G+\gamma N_{l}^{-1}G_{x^{*}}^{-1}G_{y}K-\frac{1}{2}aG_{x^{*}}^{-1}G_{h^{s}}h^{s}\\ \gamma N_{l}^{-1}K-K_{y^{*}}^{-1}K_{h^{l}}H_{L}\\ -\frac{1}{2}ah^{s} \end{array}\right] \end{array}$$

and

$$\begin{array}{c} U_{2}=\left[\begin{array}{c} a^{\frac{1}{2}}G_{x^{*}}^{-1}G_{h^{s}}\\ 0_{(N+2)\times K}\\ a^{\frac{1}{2}}I_{K} \end{array}\right], \end{array}$$

$\ddot{W}$ is a ((L+1)+(N+2)+K)-dimensional complex Wiener process.

We define the Lyapunov function of u as $V=\frac{1}{2}u^{T}u$. Based on Lemma 1, the Lyapunov drift of the dynamic system in (40) can be computed as

$$\begin{array}{ccc} LV(u)\;\;\;\;\;\;\;\;\; & & =\;\gamma N_{s}^{-1}\;x_{e}^{T}G\;+\;\gamma N_{l}^{-1}\;x_{e}^{T}G_{x^{*}}^{-1}G_{y}K\;-\;\frac{1}{2}ax_{e}^{T}G_{x^{*}}^{-1}G_{h^{s}}h^{s}\\ & & \;+\gamma N_{l}^{-1}y_{e}^{T}K-y_{e}^{T}K_{y^{*}}^{-1}K_{h^{l}}H_{L}-\frac{1}{2}ah_{s}^{T}h_{s}\\ & & \;+\mathrm{tr}\left[a{\left(G_{x^{*}}^{-1}G_{h_{s}}h_{s}\right)}^{T}\left(G_{x^{*}}^{-1}G_{h_{s}}h_{s}\right)+aI_{K}\right]. \end{array}$$

Based on the stability constraint in Lemma 2, we can obtain the following theorem for the dynamic system stability.

**Theorem** **3.** *(Convergence of the two-timescale algorithm): Suppose there exists positive constants $v_{xy}$, $v_{xh}$, $v_{yh}$ and $l_{y}$ such that $∥G_{x}^{-1}G_{y}∥\;\leq v_{xy}$, $∥G_{x}^{-1}G_{h^{s}}∥\;\leq v_{xh}$, $∥K_{y}^{-1}K_{h^{l}}∥\;\leq v_{yh}$ and $K\leq l_{y}∥y_{e}∥$. If the step size parameter γ satisfies*

$$\begin{array}{c} \gamma \left(\frac{\alpha_{x}}{N_{s}}-\frac{{\left(v_{xy}l_{l}\right)}^{2}}{4\alpha_{y}N_{l}}\right)\geq \frac{av_{h}^{2}}{8} \end{array}$$

*the two-timescale algorithm can converge to the moving equilibrium point.*

**Proof.**  The proof is shown in Appendix A. □

Then, we give the following remarks.

- The convergence of the small timescale variables and large timescale variables are coupled with each other. The term $\left(\frac{\alpha_{x}}{N_{s}}-\frac{{\left(v_{xy}l_{l}\right)}^{2}}{4\alpha_{y}N_{l}}\right)>0$ is the premise condition for the convergence of two-timescale iterations. Recall that $N_{s}$ and $N_{l}$ are the adjustment parameters of the two-timescale iterations. Then, for the given certain spectrum allocation algorithm ($\alpha_{x}$,$\alpha_{y}$,$v_{xy}$,$l_{l}$), the small timescale variables should be iterated much faster than the large timescale variables in order to achieve the convergence.
- By adjusting γ and $N_{s}$, the stability of the algorithm can be improved. However, when $N_{s}$ is too small, the algorithm must iterate more times per unit of time, resulting in more iteration overhead. Excessive γ can also cause significant steady-state error O(γ) [22].

Then, the convergence error of the two-timescale algorithm is investigated in the following theorem.

**Theorem** **4.** *(Upper Bound of Convergence Error): The convergence error of the two-timescale algorithm can be given as*

$$\begin{array}{cc} \eta_{y} & \leq {\left(\frac{N_{l}}{\gamma }\right)}^{2}{\left(\frac{v_{yh}\varsigma }{\alpha_{y}}\right)}^{2} \end{array}$$

*and*

$$\begin{array}{cc} \eta_{x} & \leq \frac{N_{s}}{\gamma }\frac{2v_{xh}^{2}Ka}{\alpha_{x}}\;+\;{\left(\frac{N_{s}}{\gamma }\right)}^{2}{\left(\frac{v_{xy}l_{y}v_{yh}^{2}\varsigma^{2}}{\alpha_{x}\alpha_{y}^{2}}\frac{N_{l}}{\gamma }\;+\;\frac{av_{xh}\pi }{2\alpha_{x}}\right)}^{2}. \end{array}$$

**Proof.**  Please refer to Appendix B for the proof. □

From Theorem 4, we can give the following observations.

- For the convergence error, $\eta_{y}$ is determined by the large timescale algorithm adjustment parameters $\left(N_{l},\gamma \right)$ and the global CSI parameter ς. However, the convergence error of the small timescale algorithm $\eta_{x}$ is affected not only by the small timescale parameters $N_{s}$, γ, *a* and π but also by the large timescale parameters $N_{l}$, γ and ς.
- For the static CSI, where the CSI parameters a=ς=0, we have $\eta_{x}=\eta_{y}=0$. Under static $h^{l}$, where ς=0, we obtain that $\eta_{y}=0$ and that $\eta_{x}$ is the same as Theorem 2. Hence, the overall convergence error only comes from the small timescale iterations.
- Under time-varying $h^{s}$ and $h^{l}$, one can increase the step size γ and decrease the timescale adjustment parameter $N_{s}$ and $N_{l}$ to reduce the convergence error at the price of larger steady-state error O(γ), larger signaling overhead and computational complexity.

### 6.3. Signaling Overhead

For the one-timescale spectrum allocation algorithm, it needs the order of $O(1/N_{s})$ iterations at the small timescale and $O(1/N_{l})$ iterations at the large timescale during each slot of the CSI. The spectrum allocation has been decomposed into two levels of problems, which are separately solved by the cloud server and network (WiFi/LTE-U), respectively. Hence, the cloud server needs to acquire the global CSI at the large timescale, and the network needs to acquire the local CSI at the small timescale. With the average signaling overheads $c_{1}$ and $c_{2}$ on the backhaul links and wireless links, the signaling overhead amount of the network is $O(K\left(\frac{c_{2}}{N_{s}}+\frac{c_{1}}{N_{l}}\right))$.

## 7. Simulation Results

In this simulation, we consider one WiFi and two LTE-U nodes. We assume that the devices are moving based on the widely adopted *Levy walk mobility model* [29]. From t=0, the device randomly chooses a destination and moves at a constant speed in $\left(0,v_{max}\right]$. Upon reaching the destination, the device randomly choose a new destination and speed to go on. We assume that the bandwidth of the whole unlicensed spectrum is 20 MHz. The maximum moving speed of the device is set as $v_{max}=2$ m/s. The minimum distance between the device and the network is set as $D_{min}=20$ m and the path loss exponent is set as ι=1.8 [30]. We compare our proposed algorithm with the following baseline schemes: **one-timescale optimal algorithm**: The cloud sever collects the real-time global CSI and computes the optimal spectrum allocation solution; this algorithm can be also considered as the case of an one-timescale algorithm under sufficient small $N_{s}$. **one-timescale based on statistical CSI**: In this algorithm, the optimal spectrum allocation solution is computed based on the statistical CSI at each slot of a large timescale. **Static algorithm**: The unlicensed spectrum is partitioned with static fraction.

**Figure 4:**
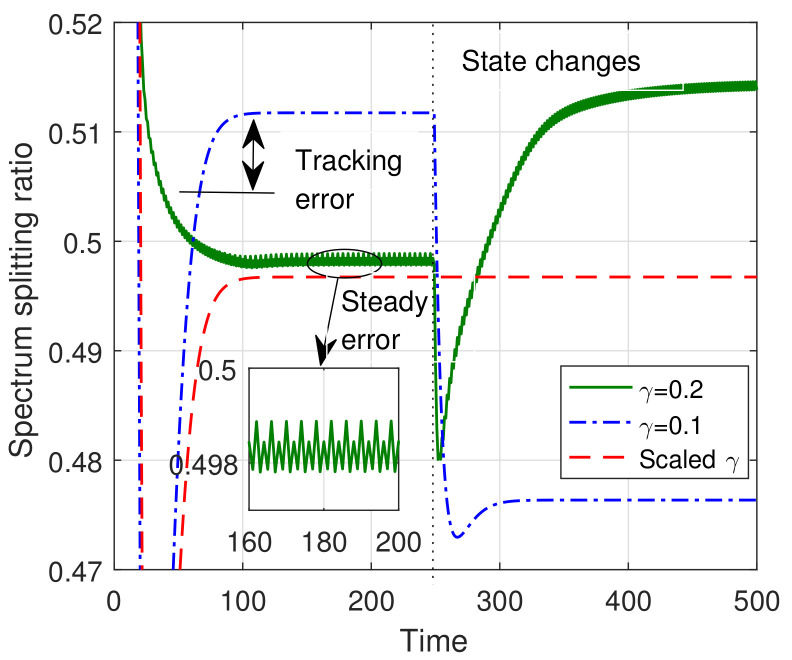
Convergence of the spectrum allocation iteration algorithm.

In the first experiment, we compared the convergence performance of the vanishing step-size and constant step-size algorithms. From Figure 4, we observe that both step size rules can make the convergence errors converge to zero under sufficient iterations. However, due to the fact that a diminishing step-size will converge to zero along with the algorithm iterations, the output solution of the algorithm will not change with the dynamics of network state. Practical, when the network state changes, the algorithm iterations should continuously approach the time-varying optimal solution. Hence, for the spectrum allocation in time-varying wireless networks, we must consider a constant step size instead of diminishing step size. The figure also shows that a large steady-error will be incurred when the step size is excessively large. This is inevitable due to the discrete iteration of the spectrum allocation algorithm.

**Figure 5:**
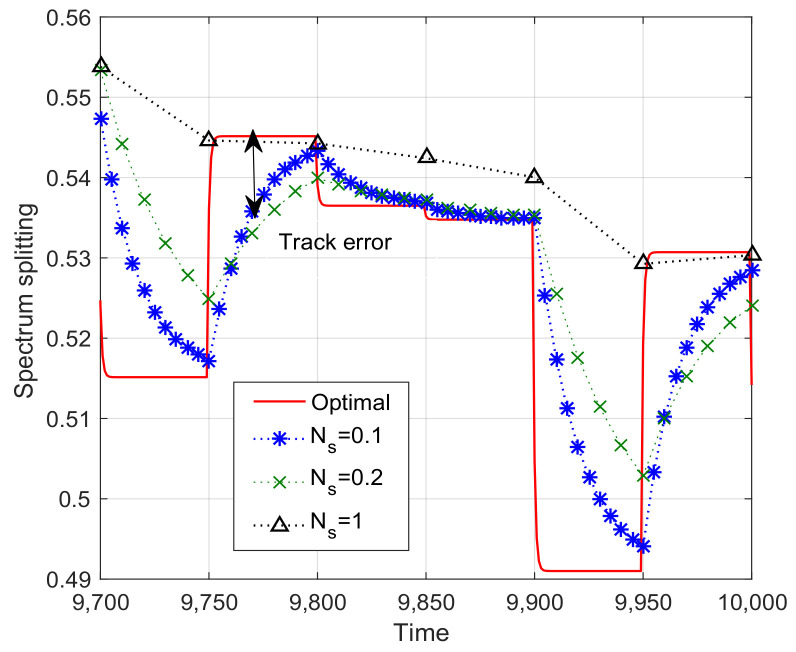
Convergence trajectory of the one-timescale spectrum allocation iteration algorithm (γ=0.01).

**Figure 6:**
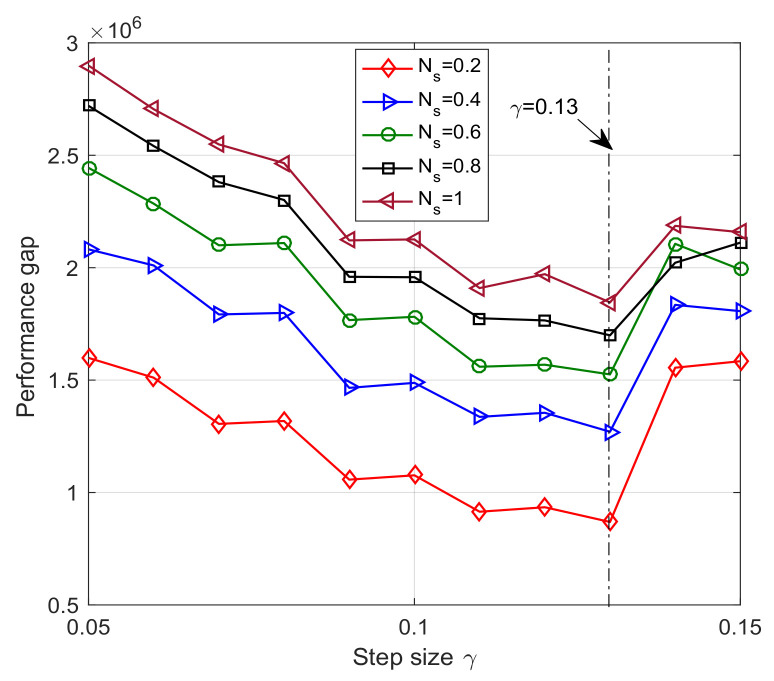
Performance gap vs. step size parameter γ.

Figure 5 illustrates the convergence trajectory of the one-timescale spectrum allocation iteration algorithm. From the figure, we observe that the small timescale adjustment parameter $N_{s}$ can help the spectrum allocation iteration algorithm converge to the optimal solution. However, as outlined in Section 4.3, the signal overhead will increase sharply with the decrease of $N_{s}$. Besides the effect of timescale adjustment parameter $N_{s}$, we investigate the relation between the step size γ and the system performance in Figure 6. The performance gap of Figure 6 is defined as the difference of weighted system throughput between the optimal algorithm and the one-timescale spectrum allocation iteration algorithm. From Figure 6, we observe that the performance gap decrease with the increase in step size parameter. However, when γ is larger than 0.13, the performance gap increase because of the steady error incurred by the constant step size. Therefore, it is not reasonable to increase the step size parameter blindly. The step size γ and timescale parameter $N_{s}$ should be jointly adjusted based on the system performance requirement and signal overhead constraint.

**Figure 7:**
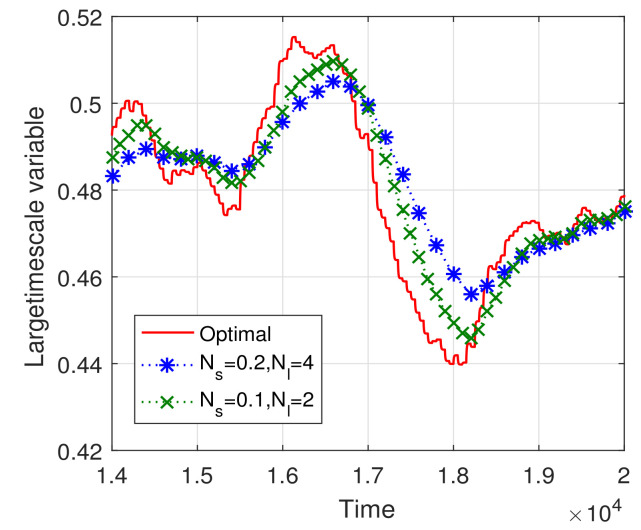
Convergence trajectory of the large timescale spectrum selection iteration algorithm.

**Figure 8:**
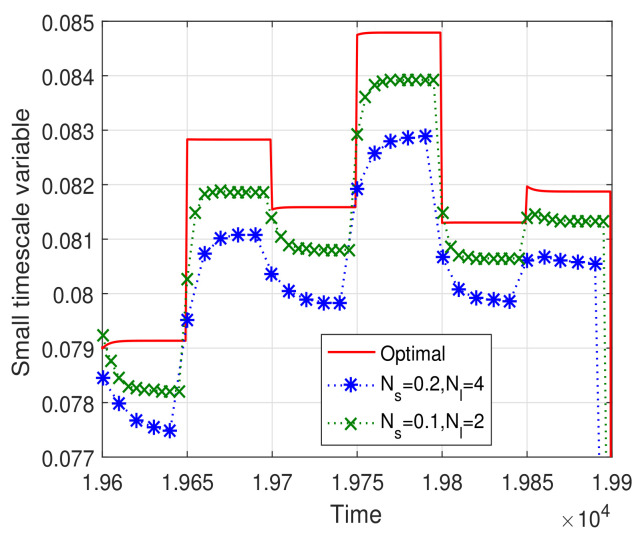
Convergence trajectory of the small timescale spectrum allocation iteration algorithm.

**Figure 9:**
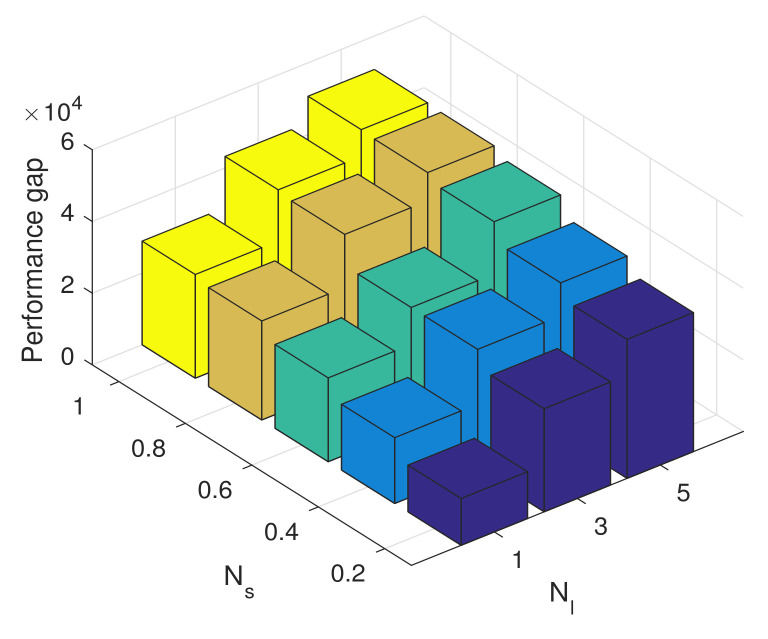
Performance gap versus $N_{s}$ and $N_{l}$.

**Figure 10:**
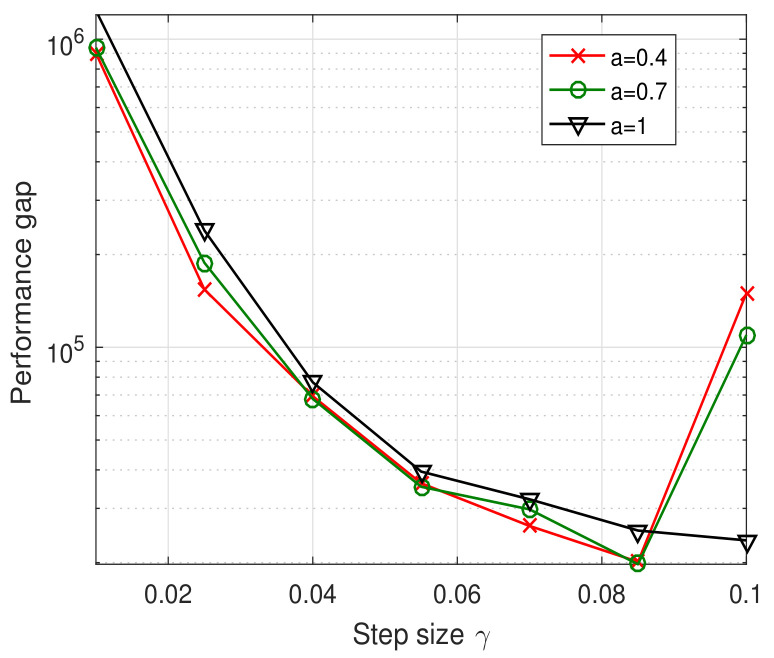
Performance gap versus *a* and γ.

Figure 7 and Figure 8 illustrate the convergence trajectories of the two-timescale spectrum allocation algorithm. We observe that the small $N_{s}$ and $N_{l}$ can help decrease the convergence error, which validates the theoretical analysis in Theorems 3 and 4. Then, in Figure 9 and Figure 10, we investigate the performance gap versus $N_{s}$, $N_{l}$. From these figures, we observe that the performance gap increases with the increase in $N_{s}$, $N_{l}$. However, the large $N_{s}$ and $N_{l}$ decrease the overhead of the two-timescale spectrum allocation algorithm. We also illustrate the relation between the step size γ and the system performance in Figure 10. Similar to the one-timescale algorithm, Figure 10 also shows that the performance gap decrease as the step size parameter increases. When the step size is larger than 0.85, a larger steady error will be incurred and the performance gap increase. Therefore, under the consideration of system performance requirement and signal overhead constraint, we can appropriately increase the step parameter γ to compensate for the performance gap of the larger $N_{s}$ and $N_{l}$.

**Figure 11:**
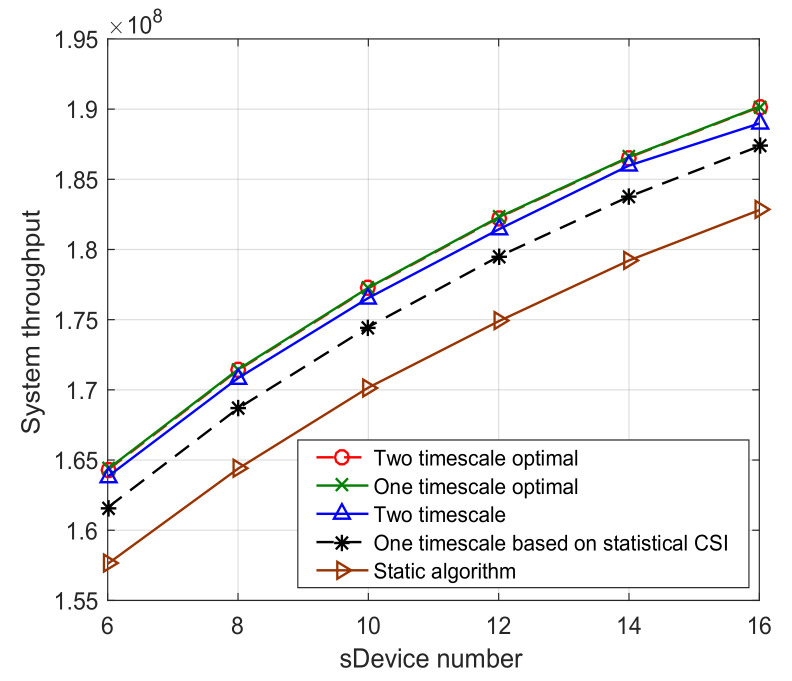
System throughput versus device number.

Figure 11 investigates the system performance versus the number of sDevices. The figure shows that the two-timescale optimal algorithm has the similar performance compared with the one-timescale optimal algorithm. This indicates the validity of decomposing the spectrum allocation into a two-timescale algorithm. That is, by decomposing the spectrum allocation into large timescale spectrum selection and small timescale spectrum allocation, the policy can achieve the maximum system throughput without the real-time global CSI. This can effectively reduce the signal overhead on both the backhaul links and wireless links. The figure also shows that although there is a little loss of system throughput of our proposed two-timescale algorithm, it performs much better than the static algorithm. Moreover, it performs better than the one-timescale dynamic spectrum allocation algorithm based on statistical CSI.

## 8. Conclusions

In this paper, we investigated the continuous-time control for the spectrum allocation in a wireless network. Specifically, we first proposed a universal convergence analysis framework for the unlicensed spectrum allocation algorithm. Then, we developed the one-timescale iteration-adjustable unlicensed spectrum allocation algorithm. For the networks with mixed timescale network sates, we proposed a two-timescale frequency allocation and derived its convergence condition and convergence error. When the user moves at high speed, the wireless channel will be affected by the Doppler shift, and it is difficult for the central server to obtain accurate CSI. In the future, we will model the uncertainty of CSI on different timescales. On the small timescale, the distribution of uncertainty is considered to be constant, but on the large timescale, the uncertainty is changing over time. According to this uncertainty model, we will design a multi-timescale algorithm for achieving the coexistence of LTE and WiFi networks.

## Data Availability

Data are contained within the article.

## Appendix A

**Proof** **of Theorem 3.** The convergence analysis in Equation (13) indicates $x_{e}^{T}G\leq -\alpha_{x}{∥x_{e}∥}^{2}$. Similarly, we can obtain $y_{e}^{T}K\leq -\alpha_{y}{∥y_{e}∥}^{2}$. Then, the upper bound of the Lyapunov drift can be computed as

$$\begin{array}{ccc} LV(z)\;\;\;\;\;\;\;\; & & \leq -\alpha_{x}\gamma ∥x_{e}{∥}^{2}N_{s}^{-1}+\gamma v_{xy}l_{y}N_{l}^{-1}∥x_{e}∥∥y_{e}∥\\ & & \;+\frac{1}{2}av_{xh}∥x_{e}∥∥h_{s}∥-\alpha_{y}N_{l}^{-1}\gamma ∥y_{e}{∥}^{2}+∥y_{e}∥v_{yh}\varsigma \\ & & \;-\frac{1}{2}{∥h_{s}∥}^{2}+v_{xh}aK+aK\\ & & \;=-\chi^{T}A\chi +b^{T}\chi +v_{xh}aK+aK \end{array}$$

where

$$\begin{array}{c} A\;=\;\left[\;\;\begin{array}{ccc} \alpha_{x}\gamma N_{s}^{-1}\;\; & -\frac{\gamma v_{xy}l_{y}N_{l}^{-1}}{2}\;\; & -\frac{av_{h}}{4}\\ -\frac{\gamma v_{xy}l_{y}N_{l}^{-1}}{2}\;\; & \gamma \alpha_{y}N_{l}^{-1}\;\; & 0\\ -\frac{av_{h}}{4}\;\; & 0\; & \frac{1}{2}a \end{array}\;\;\right],b\;=\;\left[\;\;\begin{array}{c} 0\\ v_{yh}\varsigma \\ 0 \end{array}\;\;\right] \end{array}$$

where $\varsigma =2c_{0}\iota D_{min}^{-\iota -1}v_{max}$ is the upper bound of $H_{l}$. By calculating each of the leading principle minors of A, and making them positive, Theorem 3 can be proved. □

## Appendix B

**Proof** **Proof of Theorem 4.** Firstly, we will show the convergence error of $y_{e}$. Based on Lemma 1, the Lypounov drift of $y_{e}$ can be computed as

$$\begin{array}{ccc} LV\;\;\;\;\;\;\;\;\;\;\; & & \leq \;\gamma N_{l}^{-1}y_{e}^{T}K\;-\;y_{e}^{T}K_{y^{*}}^{-1}K_{h_{l}}\varsigma \;\leq \;-\alpha_{y}\gamma N_{l}^{-1}{∥y_{e}∥}^{2}\\ & & \;+v_{yh}\varsigma ∥y_{e}∥\leq -\frac{1}{2}\alpha_{y}\gamma N_{l}^{-1}{∥y_{e}∥}^{2}+\frac{{\left(v_{yh}\varsigma \right)}^{2}}{2\alpha_{y}\gamma N_{l}^{-1}}. \end{array}$$

According to Lemma 2, the convergence error of $y_{e}$ is

(A1) $$\begin{array}{c} \eta_{y}\leq {\left(\frac{v_{yh}\varsigma }{\alpha_{y}}\right)}^{2}{\left(\frac{N_{l}}{\gamma }\right)}^{2}. \end{array}$$

For the convergence error $x_{e}$, the Lypounov drift can be computed as

$$\begin{array}{ccc} LV\;\;\;\;\;\;\;\;\;\;\; & & =\;\gamma x_{e}^{T}GN_{s}^{-1}\;+\;\gamma N_{l}^{-1}x_{e}^{T}G_{x^{*}}^{-1}G_{y^{*}}K\\ & & \;\;-\;\frac{1}{2}ax_{e}^{T}G_{x^{*}}^{-1}G_{h_{s}}h_{s}+\mathrm{tr}\left[a{\left(G_{x^{*}}^{-1}G_{h_{s}}\right)}^{T}G_{x^{*}}^{-1}G_{h_{s}}\right]\\ & & \leq -\alpha_{x}\gamma N_{s}^{-1}∥x_{e}{∥}^{2}+\gamma v_{xy}l_{y}N_{l}^{-1}∥x_{e}∥∥y_{e}∥\\ & & \;+\frac{1}{2}av_{xh}\pi ∥x_{e}∥+v_{xh}^{2}Ka. \end{array}$$

Substituting Equation (A1) into the equation above, we obtain

$$\begin{array}{ccc} LV\;\;\;\;\;\;\;\;\;\;\; & & \leq -\alpha_{x}\gamma N_{s}^{-1}{∥x_{e}∥}^{2}+v_{xh}^{2}aK\\ & & \;+\left(\frac{v_{xy}l_{y}v_{yh}^{2}\varsigma^{2}N_{l}}{\alpha_{y}^{2}\gamma }+\frac{1}{2}av_{xh}\pi \right)∥x_{e}∥\\ & & \leq -\frac{1}{2}\alpha_{x}\gamma N_{s}^{-1}{∥x_{e}∥}^{2}+C_{1}, \end{array}$$

where

$$\begin{array}{c} C_{1}=v_{xh}^{2}aK+\frac{N_{s}}{2\alpha_{x}\gamma }{\left(\frac{v_{xy}l_{y}v_{yh}^{2}N_{l}}{\alpha_{y}^{2}\gamma }+\frac{1}{2}av_{xh}\pi \right)}^{2}. \end{array}$$

Then, based on Lemma 1, the theorem can be proved. □
